# Gene Co-Expression Network Tools and Databases for Crop Improvement

**DOI:** 10.3390/plants11131625

**Published:** 2022-06-21

**Authors:** Rabiatul-Adawiah Zainal-Abidin, Sarahani Harun, Vinothienii Vengatharajuloo, Amin-Asyraf Tamizi, Nurul Hidayah Samsulrizal

**Affiliations:** 1 Biotechnology and Nanotechnology Research Centre, Malaysian Agricultural Research and Development Institute (MARDI), Serdang 43400, Selangor, Malaysia; rabiatul@mardi.gov.my (R.-A.Z.-A.); aminasyraf@mardi.gov.my (A.-A.T.); 2 Centre for Bioinformatics Research, Institute of Systems Biology, Universiti Kebangsaan Malaysia (UKM), Bangi 43600, Selangor, Malaysia; p112032@siswa.ukm.edu.my; 3 Department of Plant Science, Kulliyyah of Science, International Islamic Universiti Malaysia (IIUM), Jalan Sultan Ahmad Shah, Bandar Indera Mahkota, Kuantan 25200, Pahang, Malaysia

**Keywords:** bioinformatics tool, crop, database, gene co-expression network, transcriptomics

## Abstract

Transcriptomics has significantly grown as a functional genomics tool for understanding the expression of biological systems. The generated transcriptomics data can be utilised to produce a gene co-expression network that is one of the essential downstream omics data analyses. To date, several gene co-expression network databases that store correlation values, expression profiles, gene names and gene descriptions have been developed. Although these resources remain scattered across the Internet, such databases complement each other and support efficient growth in the functional genomics area. This review presents the features and the most recent gene co-expression network databases in crops and summarises the present status of the tools that are widely used for constructing the gene co-expression network. The highlights of gene co-expression network databases and the tools presented here will pave the way for a robust interpretation of biologically relevant information. With this effort, the researcher would be able to explore and utilise gene co-expression network databases for crops improvement.

## 1. Introduction

Transcriptomics is the measurement of transcript expression levels in different tissues, stages or conditions. In plant sciences, transcriptomics is increasingly being used to understand the expression patterns in different tissues and conditions, and to unravel the molecular mechanisms controlling biological processes [1,2,3]. The expression patterns of a gene across different tissues, development stages and conditions provide insights into when and where a gene is required, as well as clues for the gene’s function. Innovations in microarray technologies, high-throughput RNA sequencing (RNA-seq) platforms and computational biology have facilitated large-scale studies on gene expression and have led to the accumulation of transcriptomic data.

Several public databases, such as GEO [4], ArrayExpress [5] and ENA (https://www.ebi.ac.uk/ena/browser/home, accessed on 10 March 2022), have allowed researchers to upload transcriptomic data, enabling data reproducibility for others. Since then, the number of microarray and RNA-seq experiments in the GEO, ArrayExpress and ENA has grown rapidly. In recent years, there has been an increasing interest in using public microarray and RNA-seq datasets to perform gene co-expression network analysis (GCN) [6,7,8].

Gene co-expression network (GCN) provides an essential tool for the study of systems biology. The GCN demonstrates that genes are nodes, while edges represent the genes that connect to each other via co-expression relationships [9]. Co-expression shows a similarity of gene expression patterns across various experimental conditions, suggesting the function of the characterised genes to be assigned with uncharacterised neighbours in the network [9]. In addition, a GCN is described as a scale-free topology, meaning that all nodes in the network are significantly correlated with correlation coefficients above a certain threshold [10]. The GCN is widely used to deduce the unknown genes by validating known gene functional functions and regulatory relationships between genes, because genes under the same regulatory control tend to be functionally related [11,12].

**Figure 1:**
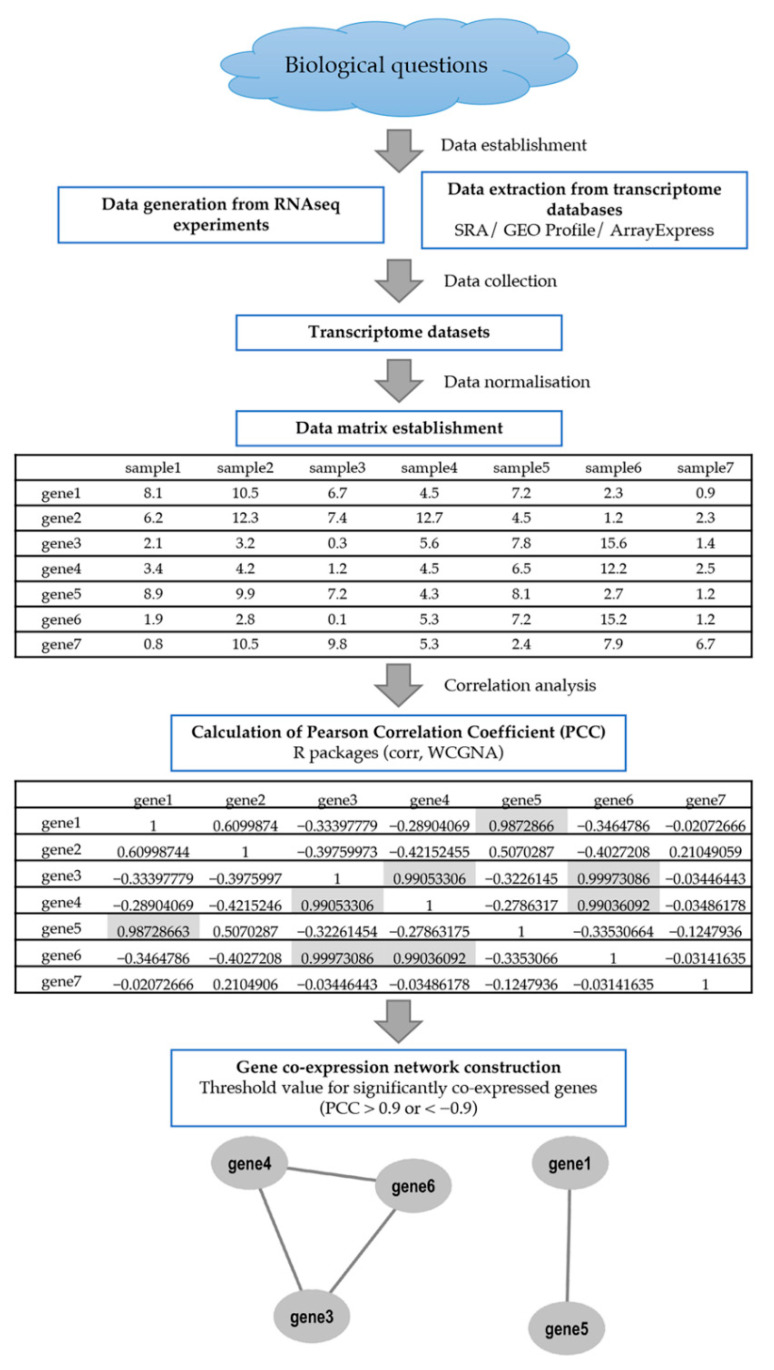
Summary of the gene co-expression network analysis pipeline. A co-expression network study is usually initiated by a biological question that would affect the experimental design of the RNA-seq and microarray experiments. The gene expression data can also be retrieved from transcriptome databases, i.e., SRA, GEO Profile and ArrayExpress. First, normalisation will be performed on the input transcriptome datasets. The generated data matrix comprises columns containing different samples and rows corresponding to genes. Next, the correlation analysis using the Pearson’s Correlation Coefficient (PCC) will be performed to calculate the degree of similarity between the gene expression profiles. Finally, the undirected GCN construction will calculate the whole gene pairs in the data matrix. The selected threshold value calculated by PCC to infer significantly co-expressed genes is >0.9 or <−0.9, highlighted in grey.

The construction of the GCN requires large-scale and high-quality datasets extracted from RNA-seq or microarray datasets, with many processing steps and a significant amount of computational resources being utilised (Figure 1). The larger the sample size, the greater the statistical significance of the relationship between genes [10].

A GCN is constructed from gene expression data in the form of a matrix, in which rows correspond to genes and columns correspond to samples. The relevant R packages (e.g., corr, WCGNA) can be used to construct the network. The Pearson correlation coefficient (PCC), Spearman’s correlation coefficient (SCC), Kendall rank correlation coefficient (KCC) and mutual information (MI) are widely used to measure the correlation between co-expressed genes [13]. The PCC measures the correlation between a pair of expression profiles while MI attempts to measure sthe tatistical dependence between two variables [14]. A threshold for correlation value (r) will be imposed to identify significantly correlated pairs. A co-expression module corresponds to a densely interconnected module that is enriched with specific biological functions [10]. To elucidate the biological functions in respective modules, gene ontology and pathway enrichment analysis could be performed.

**Figure 2:**
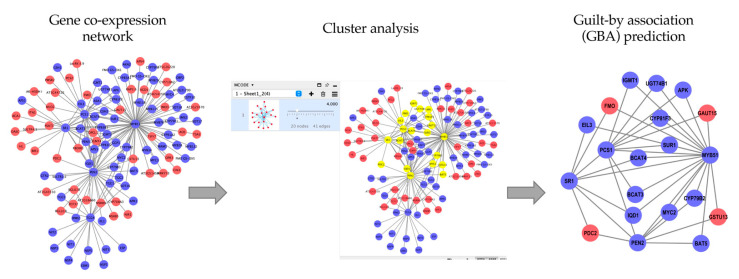
The application of GBA in identifying potential genes. First, a correlation analysis will be calculated to determine the co-expressed genes. Then, the generated GCN will be used in the clustering analysis using clustering tools, such as, MCODE, to extract the densely connected regions (yellow nodes). The GBA approach can elucidate the potential genes (red nodes) with the co-expressed known genes (blue nodes). The blue nodes are known to be involved in glucosinolate biosynthesis, which can be used to infer the red nodes as potential genes in glucosinolate biosynthesis.

Although the whole genome sequence in crops is widely developed, plant gene annotation is still scarce. Previous studies have used the GCN as a source to annotate unknown and uncharacterised genes involved in specific biological roles [15,16] and to identify new genes involved in biological mechanisms [17,18,19,20,21,22]. This is due to the hypothesis that groups of genes that are co-expressed in a module may share a similar biological function [13]. This principle follows the ‘guilt-by-association’ (GBA) method that is key in analysing the constructed GCN in functional genomics studies [23]. The summary of GBA application in identifying the unknown or uncharacterised gene in certain biological functions is shown in Figure 2.

To date, several web-based tools have been developed to allow researchers to construct the GCN, such as CoExpNetViz [24], Large-Scale Transcriptome Analysis Pipeline (LSTrAP) [25], CEMiTool [26] and CoExp [27]. In addition, in line with the RNA-seq data, GCN tools and several GCN databases have also been developed. Developing a GCN database provides a source of functional annotation for plant genes to help in the experimental elucidation of gene function, which is often laborious. Furthermore, we can combine the networks from multiple species and perform comparative networks once the GCN is established. For instance, comparing the GCN from tomato and potato has resulted in the discovery of gene modules related to steroidal glycoalkaloids [28].

In the past few years, several reviews on the GCN in plant biology have covered the topics of protocols, tools and algorithms used for performing GCN analysis. In addition, the database resources used, issues in performing statistical tests and the challenges of GCN have also been discussed comprehensively [9,13,14,29,30]. However, GCN-related databases and tools have only recently been emerged. Considering the recent advances in the GCN and transcriptome public databases, this review focuses on the recent updates on major gene co-expression tools and databases, such as their features and usefulness. We also discuss how these tools and databases can be used for comparative GCN analysis across various crop species and the current gaps in knowledge of GCN.

## 2. Overview on Co-Expression Tools for Analysing the Gene Co-Expression Network in Crops

Co-expression tools have been widely used in plant research to construct GCNs and to visualise co-expressed genes. Table 1 shows the list of available co-expression tools to construct the GCN and analyse the interaction networks in order to unravel the biological mechanism as well as to annotate unknown genes in different species of crops.

### 2.1. Web-Based Tool to Construct Gene Co-Expression Network

#### 2.1.1. CORNET

CORrelation NETworks (CORNET) is a web-based tool for constructing and visualising co-expressed genes gathered from microarray data, protein–protein interactions, and regulatory interactions [37]. The microarray datasets were retrieved from the TAIR10 databases (e.g., abiotic stress, AtGenExpress, flower and root). To date, CORNET has been developed for GCN in Arabidopsis thaliana (CORNET 2.0) and maize (CORNET Maize 1.0). Several functionalities have been developed, such as the ‘Co-expression tool’, ‘Browse Experiments’, ‘PPI tool’ and ‘TF tool’. In the ‘Co-expression tool’, users can input their genes of interest, and the correlation coefficients can be calculated using PCC or Spearman. After constructing the gene co-expression network, the gene network output will be formatted in tab-delimited or Cytoscape format. Users can visualise the network in Cytoscape.

CORNET also has other features, including “User-defined”, that allow users to upload their own microarray dataset and analyse it in the CORNET. The function under ‘PPI tool’ enables users to integrate the co-expression network with the protein–protein interaction network (PPI). Integrating the two different data types provides the flexibility to investigate the association between genes and proteins, and to obtain the functional annotation of the uncharacterised genes.

#### 2.1.2. CoExpNetViz and PlaNet

CoExpNetViz and PlaNet are co-expression tools that can perform comparative co-expression networks within and between crops species. CoExpNetViz is a co-expression tool that analyses queries of genes from transcriptome data and calculates a correlation matrix for plant species [24]. Then, it performs comparative co-expression networks and visualises the interaction networks. This co-expression tool calculates the correlation matrix using MI and PCC. When compared to other co-expressed-based tools that are mainly developed for model organisms, CoExpNetViz accepts transcriptomic data from any plant species that the users provide. This co-expression tool searches for the genes and finds the bait genes’ orthologs. It uses the concept of orthologs to identify the conserved co-expression relationships via co-expressed genes within one species and then groups the networks across multiple species. The output format will be in Cytoscape format. Additional functionalities include network hub clustering, gene ontology enrichment and network analysis.

PlaNet (Plant Network) is a comparative tool for co-expression networks of 11 plant species (i.e., A. thaliana, Hordeum vulgare, Medicago truncatula, Triticum aestivum) [30,38]. This tool performs comparative network algorithms to estimate the similarities between networks. It can predict gene function, prioritise genes and identify conserved and duplicated gene modules between the plant networks. PlaNet offers features such as ‘Network Comparer’ to compare and display similarities and differences between the co-expressed node or compare co-expression networks across the plant species. Additionally, users can perform clustering for co-expression networks in the PlaNet. The Heuristic Cluster Chiseling Algorithm (HCCA) has assigned genes into respective clusters. PlaNet features, such as the ‘famNet database’, can visualise the functional association between gene families and identify duplicated or conserved biological processes between species.

#### 2.1.3. RECoN

The Rice Environment Co-expression Network (RECoN) is a co-expression tool designed to identify clusters of functional genes that are tightly co-expressed in a collection of rice abiotic stress gene expression datasets from a wide range of environmental conditions [31]. RECoN retrieves 414 gene expression datasets from NCBI GEO and ArrayExpress databases, performs the GCN analysis and uses a graph-clustering algorithm to cluster the densely connected modules. Based on the densely connected modules, it suggests deregulated functional and regulatory mechanisms. The clusters are then linked to GO terms and KEGG pathways. This web-based tool allows users to upload differentially expressed genes’ profiles and choose the *q*-value threshold to find the most highly expressed or repressed clusters. Using RECoN will facilitate users to identify potential regulatory genes, biological processes and pathways that are crucial for abiotic stress responses.

#### 2.1.4. webCEMiTool

webCEMiTool is an online platform that enables users to perform GCN analysis for any organism. The functional studies performed in webCEMiTool include the identification of hub genes for each gene network, gene ontology and pathway enrichment analyses [26]. webCEMiTool also allows users to integrate transcriptomic data with protein–protein interactions. Users can upload gene expression data, phenotypic information and gene–gene interactions in tab-delimited format. Users can choose two correlation parameters, either Pearson or Spearman, from the drop-down list. The output is then prepared using the Cytoscape format. Previously, CEMiTool was developed based on the Bioconductor R-package. Using webCEMiTool facilitates users lacking in computational experiences to perform co-expression network analysis.

### 2.2. Command-Line Tools

#### 2.2.1. Weighted Correlation Network Analysis (WGCNA)

The weighted correlation network analysis (WGCNA) is the earliest R package for constructing the correlation network. WGCNA is built on the concept of a scale-free network where it uses a series of correlations to identify groups of genes that are expressed together in a dataset. It can find clusters of highly correlated genes, summarise the clusters using the module eigengene or an intramodular hub gene, and calculate topological properties [32]. Other functions in WGCNA include data simulation, data visualisation and comparison across modules of different species. Although the WGCNA requires command-line skills, many comprehensive tutorials in using this package are available online. A previous study has found that WGCNA is more sensitive to noise in datasets and outperforms other clustering methods in detecting non-overlapping clusters or modules [39]. The WGCNA has been reported in several publications related to crop improvement [40,41,42,43,44].

#### 2.2.2. Petal

Petal is one of the R packages developed to construct scale-free GCN models by following the standard flow of co-expression analysis [33]. Using petal, it can detect modules and identify highly connected subnetworks. petal is written in R language; thus, it requires little prior knowledge of R programming and network science or graph theory. However, petal requires fewer input parameters, making it easy for users to cater to this library. The co-expression relationships are measured using PCC, Spearman’s Correlation Co-efficient, Kendll Rank Co-efficient, Euclidean Distance, Manhattan Distance, Canberra Distance and MI. The output can also be imported into Cytoscape for network visualisation.

#### 2.2.3. LSTrAP

Handling large-volume of expression data is quite challenging. Hence, a Large-Scale Transcriptome Analysis Pipeline (LSTrAP) was developed to combine the essential bioinformatics tools to construct a GCN [25]. The process in LSTrAP includes mapping the short reads to the genome, performing quality control and constructing a co-expression network. The process starts with raw RNA-seq data until the co-expression clusters or modules are detected. In the GCN analysis, LSTrAP uses PCC for measuring correlation values among the paired-genes, while for gene clustering it uses the Markov cluster algorithm (MCL). Interestingly, LSTrAp includes functional analysis and comparative network features, for instance, by annotating the genes using InterProScan and running the orthologous genes for unknown function gene prediction. To use LSTrAP, users must download the source code from Github and perform the GCN pipeline installation. However, several bioinformatics tools (i.e., Bowtie2, TopHat, HISAT2, Samtools, Trimmomatic and MCL) that are required for RNA-seq analysis must be installed first.

#### 2.2.4. COGENT

Consistency of Gene Expression NeTworks (COGENT) is designed to facilitate users who are often unclear on the methods and parameters in the GCN analysis that should be selected. Hence, COGENT can be used to choose different co-expression measures, cut-offs and threshold choices in GCN analysis [34]. When compared to other tools, COGENT is not meant to construct co-expression networks but is aimed to evaluate them. For instance, COGENT can prioritise different network models (Pearson vs. Kendall correlation coefficient) and inform the co-expression cut-offs. It has been demonstrated that the network construction techniques prioritised by COGENT capture more protein–protein interaction data than methods that are not prioritised [34]. COGENT is an R package, and its code can be run from a terminal, RGUI and RStudio. COGENT is easy to install and use as the tutorial is comprehensive and easy-to-follow.

#### 2.2.5. GWENA

GWENA is an R package through Bioconductor that has been developed for gene co-expression network construction and analysis [35]. The GCN analyses include characterising modules, differential co-expression networks, gene connectivity, hub gene detection, gene set enrichment, phenotype association and network visualisation. GWENA was developed due to the lack of available tools that could combine the network analyses into a single pipeline, especially on differential co-expression network analysis. Both normalised RNA-seq and microarray datasets can be used as input data. The identification of the top hub gene using GWENA is based on several methods, including highest connectivity, superior degree and Kleinberg’s score.

#### 2.2.6. Juxtapose

There are not many tools available for differential co-expression network analysis. Hence, Juxtapose was developed to address this gap. Juxtapose was performed in command line mode using Python [36]. Juxtapose compares multiple co-expression networks in different conditions or tissues in the same species using a gene-embedding-based approach, which requires a local and global measure of similarity between networks based on topological networks. It has been stated that Juxtapose does not require gene orthology or variable pathways as parameters. This tool requires basic user installation using virtual machines and AWS instances.

## 3. Overview of Current Gene Co-Expression Network Databases in Plants

A total of 14 GCN web-based databases host co-expressed genes in different crop species (links and references described in Table 2). Crop breeders and researchers can rely on the resources provided by these databases to be used for crop improvements.

### 3.1. Oryza sativa

The Rice Expression Database (RED) and Rice Functionally Related Gene Expression Network Database (RiceFrend) are two co-expressed gene databases in rice. RiceFrend is a gene co-expression database based on an extensive collection of 24 transcriptome datasets representing 815 microarray datapoints, derived from various tissues and organs at different stages of growth and development under natural field conditions [46]. All the datasets were retrieved from the NCBI Gene Expression Omnibus (GEO). The RiceFrend provides a platform for identifying functionally related genes in various biological pathways. Users can use the ‘single guide gene’ function to search for co-expressed genes using a single guide gene. The feature ‘Multiple guide genes’ can be used to search for multiple genes simultaneously. The search box requires a rice gene identifier, gene name or transcription factor name from RAPDB and MSU databases. The co-expressed genes will be displayed in a table format with several descriptions, including gene description, gene symbol and the gene identifier from the RAPDB and MSU. The Mutual Rank (MR) value and HyperTree graphical viewer are displayed in the co-expressed column, which shows the relationship of co-expressed genes, weighted PCC and MR value. The co-expressed data demonstrate only the top 100 co-expressed genes. The download options are also available for users to open it in Cytoscape or Graphviz format.

RiceFREND also provides gene enrichment and cis-elements analyses. Identifying cis-elements for co-expressed genes could provide additional information for gene function prediction. Users can select the respective genes in the table box and choose the option button for each analysis to perform this analysis.

The RED database serves co-expressed data that was analysed using high-quality RNA-seq datasets obtained from NCBI SRA [45]. Seventeen RNA-seq projects have been used to construct the expression profile and co-expressed data. For the co-expressed data search, users can use the search box to query multiple genes for a maximum of 100 genes. The gene identifier can be from both the RAPDB or MSU databases. Then, users can choose the parameter of Pearson’s from 0.1 to 0.9. The results will be displayed in table and gene network format. The co-expressed genes and their pairs will be shown in the table, including PCC and *p*-value. The query gene is highlighted in red for the gene network, while its paired-gene is highlighted in blue. The results can be formatted in Excel and CSV formats and viewed in Cytoscape. In total, 11,153,091 co-expressed genes are stored in the RED database.

### 3.2. Zea mays

MCENet (maize conditional co-expression network) is a database for global and conditional co-expression network for maize [47]. The ten global and conditional co-expression networks have been generated on 701 transcriptomic and 108 epigenomic datasets. The five network tools include ‘Network Search’, ‘Network Remodel’, ‘Module Finder’, ‘Network Comparison’ and ‘Dynamic Expression View’. This database serves the maize research community to identify maize functional genes or modules that regulate the essential agronomic traits. MCENet can be accessed via http://bioinformatics.cau.edu.cn/MCENet/ (accessed on 10 March 2022). Users can use the ‘Network Search’ function to search for co-expressed genes with one or several genes. In addition, users can identify gene modules using ‘Module Finder’ and obtain the GO enrichment analysis.

Public RNA-seq maize datasets have also been used to construct the GCN for the maize GCN web-based database (http://www.bio.fsu.edu/mcginnislab/mcn/main_page.php, accessed on 11 March 2022) [6]. Three methods have been used for the GCN analysis: normalisation, network inference, and ranked aggregation. This web-based database is easy for users to explore. Users can query gene names in maize, and a table will be displayed to show a list of paired genes and their descriptions. The output data can be exported as CSV and SIF files for graph viewers.

### 3.3. Sorghum bicolor

Sorghum functional genomics database (SorghumFDB) was established as a functional genomics data mining platform [48]. This web-based database integrates gene family classifications in sorghum, miRNA, protein–protein interaction and co-expression data. The section on co-expression data consists of 144,901 positive pairs and 136,596 negative pairs of co-expressed genes in sorghum, and allows users to search for 987 modules containing 3954 co-expressed genes. The co-expressed genes are constructed from RNA-seq and microarray datasets, including different tissues (shoot, root, leaf and stem) and sorghum lines (i.e., R159, Atlas, Fremont). The correlation value is generated using the PCC score, while MR is used to calculate prediction efficiency. The visualisation of co-expressed genes can be exported into Cytoscape format. Using the SorghumFDB, the users can search the co-expressed data to understand the gene regulatory networks for sorghum improvement.

### 3.4. Vitis vinifera

*Vitis vinifera* co-expression database (VTCdb) is a web-based database used to search the gene co-expression networks of grapevine cultivars [49]. The co-expressed genes in VTCdb have been constructed using 800 publicly available microarray datasets from diverse experimental series, including 403 datasets from the Affymetrix *V. vinifera* GeneChip (16 K) and the 463 datasets from the NimbleGen Grape Whole-genome microarray chip (29 K). Four features are available in VTCdb, including ‘single guide gene query’, ‘multiple guide gene queries’, ‘keyword query’ and ‘browse meta-network. Browsing the ‘Meta-network’ feature will display the grapevine meta-network and modules of densely connected nodes. Under the ‘single guide gene query’, users can insert a grapevine gene ID into the CoexQuery field and select the predefined conditions, such as ‘All’, ‘Berry’ and ‘Stress’. The co-expression measure can also be chosen, such as HRR, MR and PCC. The result page shows the functional annotation of query genes and a list of co-expressed genes sorted by the ascending metric of interest. The information of co-expressed genes includes the gene names, probesets, modules, expression profiles and gene ontology terms. All results are displayed in a table format. The result page shows an interactive visualisation of the gene co-expression network. VTCdb also provides an analysis tool to query the expression profile for the genes of interest. Users can input the RefSeq identifier and also perform keyword searches. Mining the co-expressed genes in VTCdb facilitates users to gain insights into grapevine transcriptional regulation, gene prioritisation and the comprehensive annotation of functions for unknown genes.

### 3.5. Solanum lycopersicum

CoxPathDB is a GCN database for tomatoes, enabling the identification of strongly co-expressed genes associated with biological processes and pathways [50]. This pathway information can be used to infer the relevant pathways to a query gene and to assist in predicting the gene functions. The co-expressed genes in CoxPathDB were generated from 1234 RNA-seq datasets in the SRA database. The PCC was used to measure the co-expression relationships of paired genes. The co-expressed genes were ranked based on gene set enrichment results, followed by the degree of co-expression and over-representation analysis (ORA). In CoxPathDb, users can search for the query gene using a search box and infer the relevant pathway, which will assist in predicting gene functions. If users search using keyword terms, the search results display the list of genes IDs related to the keyword terms. Then, users can then click the gene ID to search for more details on the ranked co-expressed genes information, including the KEGG pathway name, KEGG ID, *p*-value and the *p*-score representing the ORA and GSEA analyses.

### 3.6. Malus domestica

The AppleMDO database consists of co-expressed genes analysed from 112 RNA-seq datasets of Golden Delicious apple [19]. The gene co-expression networks of apple were constructed as a global network from different developmental stages, stress treatments and tissues. A conditional co-expression network has also been constructed using 81 samples from tissue without prior stress treatment. PCC was used to measure the expression correlation between genes, and MR was used to rank the genes. AppleMDO enables the identification of co-expressed genes by providing specific gene functions and exploring the possible regulatory mechanisms of genes. The sections in the AppleMDO database include a search function for one gene or a multiple gene list.

Interestingly, AppleMDO also provides comparative co-expression networks between different species, allowing for the identification of orthologs via GCN analysis. Additional tools in the AppleMDO include gene ontology enrichment analysis, blast analysis, motif analysis, ID conversion and sequence extraction. Using AppleMDO provides molecular resources that could benefit apple research communities and serve as a reference for other fruit species.

### 3.7. Phyllostachys edulis

BambooNET is a co-expression network database of moso bamboo (*P. edulis*) that allows users to search for co-expressed genes and modules [51]. It can also perform cis-element analysis and gene set enrichment analysis of the co-expressed genes. The GCN has been constructed into global and conditional networks using 78 transcriptome datasets. This database aims to provide co-expressed genes that can be applied for improving/refining bamboo gene annotation, in order to identify the functional genes or modules and reveal the relationships between gene expression and traits of interest.

### 3.8. Camelia sinensis

TeaCON is a gene co-expression network database for tea plants (*C. sinensis*) that allows users to search for candidate genes related to agronomical traits [52]. The gene co-expression networks of the tea plant have been derived from 261 high-quality RNA-seq experiments that consisted of a wide range of tissues and treatment conditions of the tea plant. This database consists of 7,347,994 co-expressed gene pairs, covering 94% coverage of the constructed genome. TeaCON has adopted network properties, such as modularity and network density, as criteria for the cut-off in the network construction. Only the PCC with a cut-off 0.7 were deposited in TeaCON, as it considers these co-expressed genes as significantly co-expressed. Users can retrieve co-expressed genes with PCC and p-values, gene information (i.e., gene ID, description, GO and KEGG names) and co-expressed gene networks.

TeaCON has several sections, including ‘Browse’, ‘Search’, ‘Tools’ and ‘Downloads’. Users can obtain co-expressed genes in secondary metabolite pathways (i.e., theanine, caffeine and catechins) and co-expressed TF families under the ‘Browse’ sections. The results are displayed in a table format and information on the genes, including gene ID, chromosome location, gene ontology, and KEGG pathway ID, and are shown with a co-expressed gene list containing the PCC and p-values. The co-expressed genes are also visualised in a network interaction and highlighted in two different colours: red for the query genes and blue for their pairs. Format data .sif can be downloaded and opened in Cytoscape. The PCC cut-off can be adjusted from 0.6 to 1. Additional tools are integrated into the TeaCON database, such as BLAST, GO and KEGG. The resources in TeaCoN can assist the tea plant research community in understanding biological mechanisms and validating potential genes for commercial tea cultivation and characteristics.

### 3.9. Brassica napus

BrassicaEDB (https://brassica.biodb.org/, accessed on 13 March 2022), a resource for gene expression profiles of rapeseeds, and has a section on the gene co-expression networks of rapeseeds, which were constructed using WGCNA from 103 rapeseed (*B. napus* cv. ZS11) transcriptome datasets [53]. Only the top 100 strongly co-expressed paired genes with the highest weight values, PCC > 0 and *p*-value < 0.01, are deposited in BrassicaEDB. The co-expressed data can be accessed via the ‘Co-expression’ section, which provides the information on gene weight, PCC and *p*-value. A network interaction of paired genes is displayed, and it enables users to export the list of co-expressed genes in an Excel format. Users can limit the number of paired genes displayed by inserting a number in the empty box.

### 3.10. Multiple Species Gene Co-Expression Network Databases

#### 3.10.1. ATTED-II

The co-expressed genes of nine plant species—*A. thaliana* (thale cress), *Brassica rapa* (field mustard), *G. max* (soybean), *M. truncatula* (barrel medick), *O. sativa* (rice), *Populus trichocarpa* (poplar), *S. lycopersicum* (tomato), *V. vinifera* (grape) and *Z. mays* (maize)—can be searched via ATTED-II (https://atted.jp/, accessed on 13 March 2022), a plant co-expression database analysed from microarray and RNA-seq experiments (DDBJ and SRA databases) [55]. When compared to other gene co-expression databases, ATTED-II has adopted the MR index of gene-to-gene correlations as a co-expression measure because it has a higher predictive power for gene function than the PCC. Using ATTED-II, users can search for meta-co-expression analyses among nine species, investigate the statistical properties of the MR index and construct high-quality co-expression data. Furthermore, ATTED-II exploits the rank-based method, based on the ranks of two given genes in their mutual co-expression lists.

Four sections in ATTED-II include ‘Search’, ‘Browse’, ‘Draw’ and ‘Bulk’ and ‘Download’. The fastest way is to select the ‘Browse’ page, which displays the list of the co-expressed genes for subcellular location cis-elements, and summarises the co-expressed data. In the ‘Search’ section, users can query the specific genes of interest, and the results display the list of co-expressed genes. The co-expressed genes information includes the rank, average LS to query gene, gene symbol, gene function, gene ID from Entrez and RAPDB databases, hyperlink to KEGG pathway and expression patterns in a heat map format. Interestingly, ATTED-II allows the comparison of co-expressed genes among nine species, which is not available in other gene co-expression network databases. Additionally, ATTED-II enables users to analyse the co-expression relationships of genes under five pre-defined conditions, including tissue and development, abiotic stress, biotic stress, hormone treatment and different light regimes.

#### 3.10.2. PLANEX

The PLANt co-Expression (PLANEX) is a web-based database for co-expressed genes, enabling the functional identification from various Affymetrix microarray data, retrieved from the NCBI GEO database [54]. PLANEX uses the PCC value to measure the relationships of paired genes from eight plant species, including *A. thaliana*, *G. max*, *H. vulgare*, *O. sativa*, *S. lycopersicum*, *T. aestivum*, *V. vinifera* and *Z. mays*. PLANEX also uses K-means clustering for network clustering and selects a threshold of 0.001, as well as performs gene ontology enrichment analysis and Cohen’s Kappa to compare the functional similarity for all genes in the co-expression database. Using PLANEX, the user can determine the expression similarity and functional enrichment of input genes via co-expressed genes. Interestingly, PLANEX also performs a comparative gene co-expression network among species.

#### 3.10.3. PlantNexus

A valuable resource for a global gene co-expression network of barley and sorghum is PlantNexus, which enables users to search for co-expressed genes that infer regulatory mechanisms in biological processes [56]. The GCNs have been developed using 500 RNA-seq data sets for barley and 744 datasets for sorghum across tissues, developmental stages (i.e., leaf, root, shoot, flower, seed) and treatment conditions. This web interface also visualises the gene co-expression network. Users can use the search box to retrieve single or multiple genes. The results are displayed in a data table format, including paired gene identifier, gene description, gene ontology terms, pathway, mutual rank value and log2FPKM values in different tissues or treatments. Several sections in PlantNexus include ‘Data Table’, ‘Expression’ and ‘Network’. All the gene co-expression networks can also be imported into Cytoscape. PlantNexus can be accessed via https://plantnexus.ohio.edu/ (accessed on 13 March 2022).

#### 3.10.4. Co-Expression Network Toolkit (CoNekT)

CoNekT is a web-based platform that has been developed to provide information on gene expression data and co-expression networks in selected plant species [57], for instance, green alga, flowering plants and seed plants. Three crop species (*O. sativa*, *Z. mays*, *S. lycopersicum*) are included in the CoNekT. This web-based database allows users to search for co-expressed genes and their neighborhoods and perform comparative co-expression network analysis across different species and species-specific comparisons. CoNekT analysed the RNA-seq datasets from the SRA database. The GCN analysis used the highest reciprocal rank (HRR) metric score to measure the correlation relationships, while the heuristic clustering chiseling algorithm (HCCA) was used for clustering identification.

Three functionalities (Species, Tools and Search) are provided in the CoNekT. Users can choose ‘Species’, which displays a list of species and statistics of transcripts, profiles and networks. The easiest way is to select the species name, and it will display a list of gene IDs and gene descriptions. Users can identify the details of co-expressed genes and expression profiles by clicking the gene ID. Then, CoNekT provides the neighbourhood gene and cluster ID that belongs to the co-expressed genes.

Interestingly, users can choose different visualisation types, such as tables, charts and networks to display the co-expression results. Moreover, CoNekT is available for the user to download and install onto a local server. This function offers the benefits of searching large-scale expression data by allowing users to select crops or plants of interest.

#### 3.10.5. CoCoCoNet

CoCoCoNet serves as a web-based platform to compare co-expression networks between a diverse set of 14 species, including plants, zebrafish and humans [58]. The RNA-seq datasets were obtained from the SRA database, followed by co-expression network construction using Spearman’s correlation. When compared to Pearson’s correlation, Spearman’s correlation is a non-parametric approach that leads to the generation of results from a broader range of data. The data provided in CoCoCoNet contains 39,517 samples from the selected 14 species. Given the diverse set of species in this tool, users can easily obtain and compare the generated co-expression network between the target genes of interest. The performance of the two networks were measured using EGAD [59], which utilises each species’s GO terms to validate the functional connectivity of each gene within the co-expression network.

## 4. Case Study: Application of Co-Expression Networks in Biological Pathway Identification

**Figure 3:**
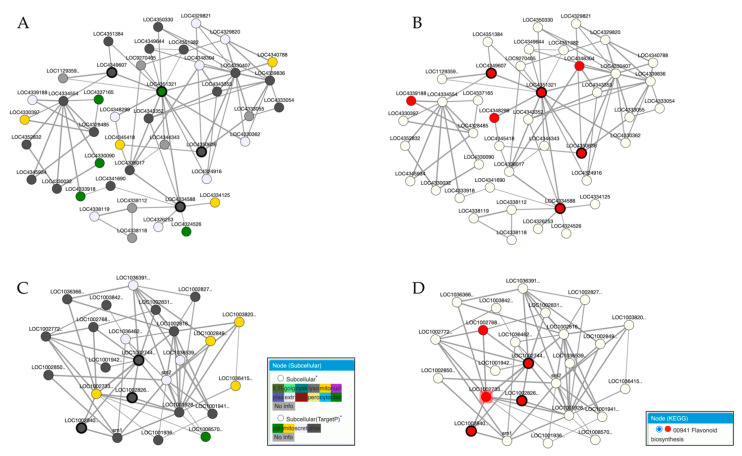
The co-expression network of CHS and CHI genes in rice (**A**) and maize (**C**). (**A**) The nodes with bold lines indicate the query genes for CHS (LOC4350636) and CHI (LOC4351321, LOC4334588, LOC4349607) in rice. (**C**) In maize, the query genes for CHS (LOC100282642, LOC100274415) and CHI (LOC100284018) are also shown with bold lines. (**A**,**C**) The co-expression network consists of genes classified based on subcellular location, calculated by TargetP. The flavonoid biosynthesis genes were selected based on KEGG in the ATTED-II database, as shown by red nodes in rice (**B**) and maize (**D**). (**D**) The highlighted gene, LOC100273383 was found to be co-expressed with known flavonoid biosynthetic genes (CHS and CHI).

To exemplify a comparative GCN analysis, we used rice chalcone synthase (CHS) and chalcone isomerase (CHI), which are parts of the flavonoid biosynthesis pathway. CHS and CHI are the key enzymes in flavonoid biosynthesis. CHS is known to catalyse the first step in the flavonoid biosynthetic pathway to produce naringenin chalcone [60]. Next, CHI catalyses the isomerisation of chalcones into (2S)-flavanones that serve as the precursor to various flavonoids in plants [61]. Rice and maize are monocots containing CHS and CHI gene families from their genomes. The first step in gene co-expression network construction is obtaining the gene ID using various databases, such as NCBI, UniProt and Ensembl. The gene IDs were obtained from NCBI Entrez Gene to provide suitable queries for the ATTED-II database in generating gene co-expression networks. Next, a gene ID for CHS (LOC4350636) and three gene IDs for CHI (LOC4351321, LOC4334588, LOC4349607) were queried into the search box in rice ATTED-II. A similar approach was conducted for maize ATTED-II using the obtained gene IDs for CHS (LOC100282642, LOC100274415) and CHI (LOC100284018). As a result, two co-expression networks were constructed, as shown in Figure 3.

Based on Figure 3, the co-expressed genes of CHS and CHI are conserved in both rice and maize, as seen by using the ATTED-II. These flavonoid biosynthetic genes are linked with the phenylpropanoid biosynthesis pathway. Figure 3D shows the example of co-expression analysis in determining an uncharacterised gene (LOC100273383) that co-expressed with the known flavonoid biosynthetic genes CHS and CHI. Thus, LOC100273383 is hypothesised to bea potential flavonoid biosynthetic gene, as supported by the KEGG database.

## 5. Perspective, Challenges and Concluding Remarks

Understanding the genotype–phenotype correlations is one of the primary issues in plant systems biology, and GCN has provided a novel avenue for researchers to investigate the interactions and associated biological mechanisms. The GCN approach has been widely used to determine whether genes are substantially co-expressed or differently co-expressed in various biological contexts. To date, the GCN has been found great utility in gene annotation not only in model systems, but also in less characterised crop species.

In this review article, we have provided an overview of available GCN tools and web-based databases. This review has revealed that each tool and database has their own uniqueness and advantages; some of them are widely used, while others are new. To make the outcome of this review, several challenges and issues in the GCN tools and databases can be addressed and suggested. We identified that six GCN tools have been developed to perform the GCN analysis using the command line approach (Table 1). This part is quite challenging for biologists as they require programming skills and a moderate level of understanding of the analysis pipeline. However, the use of command line software is suitable for analysing large-volumes of high-throughput transcriptomics datasets, which are common in the field of expression study. For non-bioinformatician or biologist users, the web-based tools are available for them to perform the GCN analysis, such as webCemiTool, CORNET 2.0, CoExpNetViz, PlaNet and RECoN.

Parameter tuning is often challenging to decide, especially in selecting the correlation threshold (i.e., range of PCC from 0.1 to 0.99). Hence, parameter optimisation is always required to reduce or avoid a bias in the GCN analysis and to generate a biologically meaningful co-expression network. The availability of web-based tools will ease the user to fine-tuning and optimise the parameters, as they can run the GCN analysis multiple times in a shorter time. 

There are four GCN tools (CoExpNetViz, PlaNet, Juxtapose WGCNA) and four GCN databases (ATTED-II, PLANEX, CoNekT and CoCoCoNet) that serve the comparative analysis of GCN across species and species-specific data. The comparative analysis of GCN can determine the similarity and differences between two or more networks. With the availability of this function, it enables users to study evolution, especially in non-model organisms [62]. For instance, several flavonoid biosynthesis genes in the genus Arabidopsis and the family Solanaceae have been discovered via comparative GCN analysis [63,64]. A previous study has demonstrated the combination of gene expression data from numerous species, allowing them to uncover potential drought tolerance genes with high levels of evolutionary-conserved regions in cereals [65].

In comparative GCN analysis, mapping or convert the gene identifier (ID) from one species to another is essential due to the different gene ID formats used. The common gene ID used in the GCN databases are from the Ensembl and Entrez databases. Users must understand the gene ID that belongs to the individual species. However, not many GCN databases offer a gene ID conversion tool. Providing the gene ID conversion tool will ease the user to convert the gene ID between various species in a single platform and in a high-throughput manner.

Among the 14 GCN databases, nine of them used public transcriptome datasets to generate the GCN. This finding indicates that there is a growing interest in using public datasets that are beneficial for crop biologists. Interestingly, the combination of microarray and RNA-seq datasets for constructing the GCN could increase the biologically meaningful information. Different statistical methods have been used to construct the GCN databases, such as PCC, SCC, Highest Reciprocal, MI and MR. For instance, PCC is often utilised across multiple databases because of its sensitivity to outliers and has been well-performed on linear relationships between two variables. However, its performance reduce on non-linear relationships [66]. SCC is less utilised as it is less susceptible to outliers by assigning ranking values, instead of utilising the expression levels itself [13]. KCC is more robust on non-normal distributions, but researchers have not chosen it due to the fact that expression level information is not considered [67].

Different databases use different strategies in generating co-expression data, i.e., how the transcriptome dataset is chosen, how gene expression is quantified and normalised and what statistical metrics are used to measure co-expression. These are all examples of discrepancies, thus giving co-expression analyses even more ways to be performed. Some of the databases (i.e., maize, SorghumFDB, VTCdb and BambooNet) used multiple statistical methods to measure the co-expression relationships, while others used only one statistical method (i.e., RED, TeaCoN). The combination of multiple statistical methods could increase the sensitivity across a multiple network structure and facilitate the decision of candidate genes for predicting their function and performing experimental validation. At the moment, there is no agreement on the best statistical methods, since different approaches work best for answering different biological questions [68].

The most widely used correlation method is PCC due to its simplicity [69]. Although PCC measures the strength of the linear relationship between two variables, it can be sensitive to outliers that may result in false correlations [70]. Furthermore, a linear relationship is not the only correlation metric observed in biological systems. Complex interactions exist in biological systems, which can be measured using non-linear relationships. Therefore, researchers should not discard non-linear relationships, as this will limit the ability to identify the accurate gene modules. Considering non-linear relationships, it will provide more or less straightforward applications in analysing gene clustering and gene regulatory networks [69]. Gini correlation coefficient (GCC) is a statistical method that employs the measurement of non-linear relationships between gene variables. The GCC assess the correlation between two variables, either in normal or non-normal distributions, and its algorithm simultaneously ranks and values the information of paired-genes, indicating it is suitable in detecting non-linear relationships as compared to other correlation methods [67], for example, to construct and infer gene regulatory network. Huang et al. [71] has introduced a count statistic (CS) method to measure non-linear relationships between paired genes in ordered and time-series samples. This statistical method is an order correlation metric and uses local information in gene expression profiles [72].

To overcome this barrier, it has been suggested to combine linear and non-linear relationships, in which the GCN analysis results will be more comprehensive [73]. By using both approaches, multiple genes and higher-order regulatory patterns can be captured simultaneously and efficiently (i.e., regulatory interactions between transcription factors) [69]. For instance, PCC coupled with MI has been used in CoExpNetViz and ATTED-II to provide the rank of co-expressed genes [24,55]. Mutual information can identify and characterise non-linear relationships since it is a generalised correlation measure [74]. Distance Correlation (DC) has also been used to measure non-linear relationships [75,76]. Although non-linear relationships are essential for complex interactions, they can be diverse, and the statistical power for detecting such relations is lower than linear-based correlation [73].

Another speciality that can be observed in the GCN database is the flexibility of the output format that can be imported into the Cytoscape. Hence, users can edit and improve the biological network of their interest. Finally, the major challenge in the GCN web-based database is updating the database regularly, based on the upcoming transcriptomic data of the future. Not many GCN databases include the ‘last-update’ on their page. Consequently, users find it difficult to know whether the co-expressed data is the latest update or not.

Gene co-expression network analysis offers an efficient approach for suggesting hypotheses in gene function prediction [23]. Researchers have to avoid from over-interpreting co-expressed data for annotating unknown genes with essential functions. Prediction of gene function could be improved via combining a GCN with different omics data, such as QTL mapping and GWAS approaches [77,78]. For example, several potential genes that affect water-stress tolerance and seed vigour have been successfully found in tomato and rice, by integrating data from GWAS, QTLs, eQTLs and differentially expressed genes [77,78].

Despite this, the ability of plant scientists to effectively adopt computational approaches is heavily dependent on database functionalities and features, such as user-friendly interfaces, simple accessibility, manuals and tutorial videos. Numerous user-friendly GCN databases could be widely utilised to aggregate omics-scale data from diverse approaches in order to annotate the candidate genes and assign hypotheses involved in specific traits. Consequently, it will improve the crop traits and increase agricultural yield and climate change resilience. Until now, only 14 GCN databases are available for some agriculturally important crops (i.e., rice, maize, sorghum, grapevine, tomato, bamboo, apple, tea and Brassica). The GCN database is still lacking in economic crops, such as banana, cocoa, durian, papaya, peach and strawberry. Hence, we expect the list to grow in the future.

## Figures and Tables

**Table 1:** List of co-expression tools for a gene co-expression network analysis in the crops.

Types	Co-Expression Network Tool	Descriptions	References
Web-based tool	CORNET 2.0 https://bioinformatics.psb.ugent.be/cornet (accessed on 11 March 2022)	An integrating tool for plant co-expression network	[29]
	http://bioinformatics.psb.ugent.be/webtools/coexpr/ (accessed on 11 March 2022)	A comparative co-expression network construction and visualisation	[24]
	PlaNet www.gene2function.de (accessed on 11 March 2022)	A tool for comparative co-expression network analyses	[30]
	RECoN https://plantstress-pereira.uark.edu/RECoN/ (accessed on 11 March 2022)	A co-expression tool to identify co-expressed genes in abiotic stress response	[31]
	webCemiTool https://cemitool.sysbio.tools/ (accessed on 11 March 2022)	A web-based tool to identify co-expression modules in a given co-expression network	[26]
	CoExp https://rytenlab.com/coexp (accessed on 11 March 2022)	A web tool for the exploitation of co-expression networks	[27]
Command-line based tool & require installation	WGCNA https://horvath.genetics.ucla.edu/html/CoexpressionNetwork/Rpackages/WGCNA/ (accessed on 9 April 2022)	An R package for performing weighted correlation network analysis	[32]
	petal https://github.com/julipetal/petalNet (accessed on 9 April 2022)	An R package for co-expression network modelling	[33]
	LSTrAP https://github.molgen.mpg.de/proost/LSTrAP (accessed on 9 April 2022)	A pipeline to construct co-expression networks from RNA-seq data	[25]
	COGENT https://github.com/lbozhilova/COGENT (accessed on 9 March 2022)	An R package to construct a gene co-expression network without the need for annotation or external validation data.	[34]
	GWENA https://bioconductor.org/packages/release/bioc/html/GWENA.html (accessed on 9 March 2022)	An R package developed to extend the analysis of gene co-expression network	[35]
	Juxtapose https://github.com/klovens/juxtapose (accessed on 9 March 2022)	A tool to compare gene co-expression networks (GCNs)	[36]

**Table 2:** Summary of gene co-expression network-related databases in publicly available crops.

Plant Species	Databases	Descriptions	Statistical Methods	References
*Oryza sativa*	Rice Expression database http://expression.ic4r.org/co-search (accessed on 29 March 2022)	A repository of gene expression profiles and co-expression network.	PCC	[45]
	RiceFrend https://ricefrend.dna.affrc.go.jp/ (accessed on 10 March 2022)	A gene co-expression database in rice based on an extensive collection of microarray data derived from various tissues/organs at different stages of growth and development under natural field conditions.	PCC & Mutual Rank	[46]
*Zea mays*	MCENet http://bioinformatics.cau.edu.cn/MCENet/ (accessed on 10 March 2022)	A database for maize co-expression networks.	PCC and Mutual Rank	[47]
	Maize gene co-expression network database https://www.bio.fsu.edu/mcginnislab/mcn/main_page.php (accessed on 10 March 2022)	A gene co-expression network database for maize.	PCC, KCC, SCC and Mutual Information	[6]
*Sorghum bicolor*	Sorghum Functional Genomics Database (SorghumFDB) http://structuralbiology.cau.edu.cn/sorghum/index.html (accessed on 15 March 2022)	A sorghum database to predict gene function.	PCC and Mutual Rank	[48]
*Vitis vinifera*	VTCdb: ViTis Co-expression DataBase http://vtcdb.adelaide.edu.au/Home.aspx (accessed on 15 March 2022)	A database for co-expressed genes in grapes.	PCC, SCC, Highest Reciprocal and Mutual Rank	[49]
*Solanum lycopersicum*	Co-expressed pathways database for tomato http://cox-path-db.kazusa.or.jp/tomato/ (accessed on 10 March 2022)	A database for co-expressed genes in tomatoes.	PCC, ORA (p-value), GSEA (p-value, percentile-scores)	[50]
*Phyllostachys edulis*	BambooNET http://bioinformatics.cau.edu.cn/bamboo/ (accessed on 10 March 2022)	A database of co-expression networks with functional modules for bamboo.	PCC and Mutual Rank	[51]
*Malus domestica*	AppleMDO http://bioinformatics.cau.edu.cn/AppleMDO/ (accessed on 10 March 2022)	A multi-dimensional omics database for apple co-expression networks and chromatin states.	PCC and Mutual Rank	[19]
*Camellia sinesis*	TeaCoN http://teacon.wchoda.com/ (accessed on 10 March 2022)	A database of gene co-expression network for tea plants.	PCC	[52]
*Brassica napus*	BrassicaEDB https://brassica.biodb.org/ (accessed on 13 March 2022)	A database of gene co-expression network and expression profiles for Brassica crops.	PCC and weight value	[53]
Multiple crop species	PLANEX http://planex.plantgenomicslab.org/ (accessed on 13 March 2022)	A plant gene co-expression database obtained from GEO NCBI.	PCC, Gene enrichment analysis (Cohen’s Kappa)	[54]
	ATTED-II https://atted.jp/ (accessed on 13 March 2022)	A plant co-expression database.	PCC, SCC and Mutual Rank	[55]
	PlantNexus http://planex.plantgenomicslab.org/ (accessed on 13 March 2022)	A gene co-expression network database for barley and sorghum.		[56]
	CoNekT-P https://conekt.sbs.ntu.edu.sg/ (accessed on 19 May 2022)	An online platform that allows users to browse co-expression networks and perform comparative GCN analysis across different crop species (rice, maize, tomato) and others plant species.	HRR and HCCA	[57]
	CoCoCoNet https://milton.cshl.edu/CoCoCoNet/ (accessed on 19 May 2022)	A comparative gene co-expression network portal for a diverse range of species including plants, humans and animals.	SCC	[58]

## Data Availability

Not appliable.
